# Use of Genotype MTBDRplus Assay for Diagnosis of Multidrug-Resistant Tuberculosis in Nepal

**DOI:** 10.1155/2017/1635780

**Published:** 2017-08-13

**Authors:** Elina Maharjan, Narayan Dutt Pant, Sanjeev Neupane, Jyoti Amatya, Bhawana Shrestha

**Affiliations:** ^1^Department of Microbiology, Kathmandu College of Science and Technology, Kathmandu, Nepal; ^2^Department of Microbiology, Grande International Hospital, Dhapasi, Kathmandu, Nepal; ^3^Central Department of Microbiology, Tribhuvan University, Kirtipur, Kathmandu, Nepal; ^4^German-Nepal Tuberculosis Project (GENETUP), Kalimati, Kathmandu, Nepal

## Abstract

The main aims of this study were to study the patterns of mutations in rpoB, katG, and inhA genes in* Mycobacterium tuberculosis* strains isolated from patients from Nepal and to evaluate the performance of genotype MTBDRplus assay, taking conventional drug susceptibility testing as gold standard for diagnosis of MDR-TB. A total of 69* Mycobacterium tuberculosis* strains isolated from 73 smear positive sputum samples from patients suspected of suffering from multidrug-resistant tuberculosis were used in our study. The drug susceptibility pattern of* Mycobacterium tuberculosis* isolated from these sputum specimens was determined by using genotype MTBDRplus assay taking conventional drug susceptibility testing as reference. The sensitivity and specificity of the genotype MTBDRplus assay for the detection of MDR-TB were found to be 88.7% and 100%, respectively. 88.7% of the rifampicin resistant isolates had mutations in rpoB gene. Similarly, 79.7% and 9.4% of isoniazid resistant isolates had mutations in katG and inhA genes, respectively. Genotype MTBDRplus assay was found to be very rapid and highly sensitive and specific method for diagnosis of MDR-TB and will be very helpful for early diagnosis of MDR-TB in high tuberculosis burden countries.

## 1. Introduction

Tuberculosis is a serious public health problem mainly in developing countries [1]. More than 95% of the tuberculosis cases and deaths related to tuberculosis occur in poorer countries [2]. Further, the multidrug-resistant tuberculosis is emerging as a serious threat to tuberculosis control programs in developing countries [2]. According to World Health Organization (WHO) report, in 2012 around 450,000 people developed MDR-TB, among which 170,000 people died [3]. Nepal is situated between two countries (China and India) with high MDR-TB prevalence [2]. According to recent drug resistance surveillance in Nepal, 2.6% of new cases and 17.6% of previously treated cases were MDR-TB positive [4].

MDR-TB is the tuberculosis caused by the strains of* Mycobacterium* which are resistant to first-line drugs: rifampicin (RIF) and isoniazid (INH), and its treatment is costly and less effective, which may result into treatment failures and relapses [3]. For the effective control of the MDR-TB, the treatment of the tuberculosis patient should be guided by drug susceptibility pattern of the strain of* Mycobacterium* causing infection in the particular patient [1]. WHO has recommended the conventional drug susceptibility testing as gold standard but, due to its long turnaround time (25 days to more than 2 months), the treatment given to the patients will be delayed resulting into higher risk of treatment failure and continuous transmission of the MDR-TB [3]. Further, the rate of contamination in case of conventional method is very high [5].

So, there is a great need of a simple but rapid method for detection of multidrug resistance among the strains of* M. tuberculosis* and its molecular basis [6]. This not only will help the clinicians for appropriate treatment of the tuberculosis patients but also will be very helpful in monitoring of the emergence of drug resistance in different geographical regions [6].

The World Health Organization approved the use of molecular line-probe assays (LiPAs) for diagnosis of MDR-TB in 2008 [3]. Genotype MTBDRplus is a line-probe assay, which can be used for the rapid detection of MDR-TB directly from smear positive sputum samples or isolates of* Mycobacterium tuberculosis* [5]. But the clinical utility of the test depends upon the type of mutations prevalent in the particular place and those targeted by the test for determination of drug susceptibility [5].

Genotype MTBDRplus assay is a rapid test which can detect MDR-TB within 8 hours and includes the steps: deoxyribonucleic acid (DNA) extraction, multiplex polymerase chain reaction (PCR), reverse hybridization, and resistance gene mutations detection [3].

Almost all rifampicin resistance is caused due to mutation in rpoB gene while mutations in the katG and inhA genes confer high-level and low-level INH resistance, respectively [6]. Genotype MTBDRplus assay detects all these mutations. In a recent meta-analysis performed by Bai et al., the pooled sensitivity and pooled specificity (95% CI) of genotype MTBDRplus assay for diagnosis of MDR-TB were 0.91 (0.86–0.94) and 0.99 (0.99–1.00), respectively [3]. But validation of the assay with respect to locally prevalent strains of* M. tuberculosis *is recommended [1]. In context of Nepal, limited data are available regarding the performance of genotype MTBDRplus assay for diagnosis of MDR-TB. So, in this study we studied the patterns of mutations in rpoB, katG, and inhA genes in* M. tuberculosis* isolates from patients from Nepal and evaluated the performance of genotype MTBDRplus assay taking conventional drug susceptibility testing as gold standard.

## 2. Methods

### 2.1. Study Design

A comparative cross sectional study was carried out from July 2012 to January 2013 at German-Nepal Tuberculosis Project, National Reference Laboratory, Kathmandu, Nepal. All 69* Mycobacterium tuberculosis* strains isolated from 73 smear positive sputum samples from patients suspected of suffering from multidrug-resistant tuberculosis were used in our study. These sputum samples were received from different DOTS Plus centers of Nepal.

### 2.2. Mycobacterial Culture of Sputum Samples

The concentrated and decontaminated sputum samples were inoculated into Lowenstein-Jensen media. And the media were then incubated at 37°C for up to 8 weeks and observed for growth every week. The colonies grown were identified with the help of acid fast staining and biochemical tests. After the identification of the* M. tuberculosis* it was harvested for drug susceptibility testing. The drug susceptibility patterns of* M. tuberculosis* isolated were determined by using genotype MTBDRplus assay taking conventional drug susceptibility testing as reference.

### 2.3. Conventional Drug Susceptibility Testing

The drug susceptibility testing of the* M. tuberculosis* isolates to first-line drugs (INH, streptomycin, RIF, and ethambutol) was performed on Lowenstein-Jensen medium by proportional method [7]. The critical concentrations of isoniazid, rifampicin, ethambutol, and streptomycin used were 0.25 *μ*g/mL, 32 *μ*g/mL, 2 *μ*g/mL, 4 *μ*g/mL, respectively. The final interpretation of the drug susceptibility testing was done after 42 days of incubation at 37°C and the presence of resistant bacilli in the proportion more than 1% indicated the strain to be resistant.

### 2.4. Genotype MTBDRplus Assay

Genomic DNA of Mycobacterial culture was extracted by incubating the colonies dissolved in 300 *μ*L of molecular biology grade water for 20 minutes at 95°C in water bath followed by 15 minutes in ultrasonic bath and centrifugation for 5 minutes at 12000 rpm [2]. Polymerase chain reaction (PCR) and hybridization were performed following manufacturer's recommendations (Hain Lifescience, Nehren, Germany) [6]. Colorimetric method (using streptavidin conjugated with alkaline phosphatase and substrate buffer) was used to detect hybridized amplicons [2]. The strip containing hybridized amplicons were interpreted following manufacturer's instructions [6].

### 2.5. Data Analysis

For the analysis of the data, statistical package for social sciences version 20.0 was used. Sensitivity, specificity, positive predictive value, and negative predictive value were calculated at 95% confidence level.

## 3. Results

Of total 73 smear positive sputum samples, 69 specimens showed the growth of* M. tuberculosis* complex while 4 specimens showed the growth of nontuberculous mycobacteria.

### 3.1. Conventional Drug Susceptibility Testing Results

Of the 69* M. tuberculosis* isolates, 35 were resistant to RIF and INH only, 27 were resistant to all first-line drugs, 5 were sensitive to all first-line drugs, and 2 were sensitive to RIF only.

### 3.2. Patterns of Amino Acid Changes and Mutations in rpoB Gene

Total 7 different hybridization patterns and six specific mutations on 81 bp hot spot region of rpoB gene were noticed among all 69 isolates tested. Further, 44 isolates showed the specific nucleotide exchange TCG to TTG in codon 531, resulting in the replacement of amino acid serine by leucine. Similarly, 2 strains had a mutation located in the region from codons 531 to 535. In addition, other mutations were noted in codon 522 in 1 strain, codon 526 in 2 strains, and codon 516 in 3 strains (Table 1).

### 3.3. Patterns of Amino Acid Changes and Mutations in katG Gene

Mutation in codon 315 of katG gene was detected in 51 isolates, of which 49 isolates had ACC (S315T1 Ser-Thr) mutation. 18 isolates had not shown any mutation pattern in codon 315 of katG gene (Table 2).

### 3.4. Patterns of Amino Acid Changes and Mutations in inhA Gene

Six isolates revealed the mutation patterns in the ribosome binding site of inhA. Among them 5 isolates had specific mutation on inhAC15T probe with omission of wild type 1 and 1 had mutation on inhAT8C probe with deletion of wild type 2, without specific mutation band (Table 3).

### 3.5. Comparison of Genotype MTBDRplus Assay and Conventional Drug Susceptibility Testing for Identification of Drug-Resistant Isolates

Of total 62 rifampicin resistant* M. tuberculosis* isolates detected by conventional drug susceptibility testing, mutation in the rpoB gene was detected in 55 isolates, while none of the 7 rifampicin sensitive isolates had mutation in the 81 bp hot spot of rpoB gene. Similarly, among 64 isoniazid resistant isolates, the mutation in codon 315 of katG gene was detected in 51 isolates but not in any of the 5 isoniazid sensitive isolates and 6 isolates had mutation on the regulatory region of inhA gene. The sensitivity and specificity of genotype MTBDRplus assay for the detection of MDR* M. tuberculosis* isolates were 88.7% and 100%, respectively. Similarly, the positive predictive value and negative predictive value for diagnosis of MDR-TB were 100% and 50%, respectively (Table 4).

### 3.6. Comparison of INH Resistance Detected by Conventional Drug Susceptibility Testing and Genotype MTBDRplus Assay

Among 64 INH resistant isolates detected by conventional drug susceptibility testing, MTBDRplus assay identified 57 strains as INH resistant and 7 strains as INH susceptible, while all of the 5 INH susceptible strains identified by conventional drug susceptibility testing were found to be INH sensitive by the assay. In comparison to the gold standard conventional drug susceptibility testing, the sensitivity and specificity of genotype MTBDRplus for detection of INH resistance were found to be 89.1% and 100%, respectively, whereas positive predictive value and negative predictive value were reported to be 100% and 41.7%, respectively.

### 3.7. Comparison of RIF Resistance by Conventional Drug Susceptibility Testing and Genotype MTBDRplus Assay

Out of total 62 RIF resistant isolates detected by conventional drug susceptibility testing, genotype MTBDRplus assay identified 55 strains as RIF resistant and 7 strains as RIF sensitive. All of the 7 RIF susceptible strains detected by conventional drug susceptibility testing were found to be RIF susceptible by the assay. In comparison to conventional drug susceptibility testing, the sensitivity and specificity of genotype MTBDRplus for detection of RIF resistance were reported to be 88.7% and 100%, respectively, while positive predictive value and negative predictive value were 100% and 50%, respectively.

## 4. Discussion

In our study, the sensitivity and specificity of MTBDRplus assay for the detection of INH resistance were 89.1% and 100%, respectively. Similarly, those for detection of RIF resistance were 88.7% and 100%, respectively. In a recent meta-analysis, Bai et al. reported the pooled sensitivity and specificity for identification of isoniazid resistance to be 0.91 and 0.99, respectively [3]. Similarly, those for identification of rifampicin were 0.96 and 0.98, respectively [3]. Further, in our study, the sensitivity and specificity of MTBDRplus assay for the detection of MDR* M. tuberculosis* isolates were 88.7% and 100%, respectively. But, according to Bai et al., the pooled sensitivity and pooled specificity of MTBDRplus assay for the detection of multidrug resistance were 0.91 and 0.99, respectively [3]. However, in a study in Ethiopia, the sensitivity and specificity of the genotype MTBDRplus assay for the detection of RIF resistance among the strains of* M. tuberculosis* were 80.0% and 99.6%, respectively [6]. Further, those for detection of INH resistance were 82.7% and 99.6%, respectively, and for diagnosis of MDR-TB were 75.0% and 100%, respectively [6]. Similarly, in a study by Huyen et al. in Vietnam, MTBDRplus detected 93.1% of rifampicin resistance, 92.6% of INH resistance, and 88.9% of MDR-TB, correctly, and specificity for each was 100% [1]. Similar sensitivity and specificity as in our study for the detection of INH and RIF resistances were also reported by another study in Nepal [2]. In this study, genotype MTBDRplus assay was performed on DNA extracted from cultures in Lowenstein-Jensen medium using the DNA extraction method we have used [2]. In addition, in a study from India, sensitivity and specificity for detection of RIF resistance were 98% and 99%, respectively; those for INH resistance detection were 92% and 99%, respectively, and 97% and 100%, respectively, for detection of MDR-TB in sputum [5]. Mutations in other genes that are not targeted by the assay may be the reason for difference in sensitivity of genotype MTBDRplus assay and that of phenotypic drug susceptibility testing [2].

In the present study, the predominant mutation for RIF resistance was found to be in rpoBS531L (71%), which was in agreement with the finding by Yadav et al. (72%) [5]. Lower rates of S531L missense mutation in rpoB gene in comparison to our study were reported by Sharma et al. (50%) [2] and Dahal et al. (37.1%) [8]. In our study, the substitution of aspartate by valine at codon 516 of rpoB gene was found in 4.8% of the RIF resistant isolates which was similar to the findings of the studies in Nepal (5.7%) [8] and India (3.%) [5]. In the present study, mutation in rpoBH526Y site was observed in 3.2% of the RIF resistant isolates. Various rates of mutations (2.8%–40%) in this region have been reported from different geographical locations [2]. However, no mutation in this region was noted by Sharma et al. [2].

Rates of mutations in katG and inhA genes are known to be different in different geographical regions [5], which were found to be in 79.7% and 10.9%, respectively, of total isoniazid resistant isolates. But Yadav et al. noticed these mutations in 83% and 11%, respectively, of isoniazid resistant isolates, with combined katG and inhA mutation in 6% of isoniazid resistant isolates [5].

In our study, mutation in katGS315T1was observed in 76.6% of INH resistant strains, which was less in comparison to another study in Nepal (90.6%) [2]. In our study mutation in the inhA promoter region (inhAC15T) among INH resistant* M. tuberculosis* strains was observed in 7.8% of isolates, which was higher than the finding of a study in Nepal (3.1%) [2] but lower than the finding by Huyen et al. (18%) [1]. In our study 1 INH resistant isolate with mutation in inhAT8C was also reported.

Genotype MTBDRplus assay is rapid and highly sensitive and specific for detection of MDR-TB directly from smear positive sputum samples or isolates of* M. tuberculosis*. Hence, it may be very helpful for early diagnosis and timely proper treatment of multidrug-resistant tuberculosis in high tuberculosis burden areas. But it does not have high sensitivity for detection of MDR-TB from samples containing low concentration of bacilli and needs trained manpower [5].

## 5. Limitations of the Study

The strains of* Mycobacterium tuberculosis* we used in our study were already isolated and stored. So, we could not find more information on the specimens from which they were isolated apart from the fact they were received from the cases of suspected multidrug-resistant tuberculosis (relapse, treatment failure, and default and chronic cases) from different DOTS Plus centers of Nepal. However, all the* M. tuberculosis* strains isolated from all the cases of suspected multidrug-resistant tuberculosis were included in our study. Further, unexpectedly low sensitivity of MTBDRplus for rifampicin resistance could be due to false-positive phenotypic drug susceptibility testing for this drug.

## 6. Conclusions

Genotype MTBDRplus assay was found to be very rapid and highly sensitive and specific method for determination of drug susceptibility patterns of* M. tuberculosis*. It is also helpful for determination of monoresistance to isoniazid and rifampicin. It will be very helpful for early diagnosis of MDR-TB in high tuberculosis burden countries, hence helping in effective implementation of tuberculosis control program and controlling the development of further drug resistance, which would otherwise have resulted into emergence of extensively drug-resistant tuberculosis (XDR-TB).

## Figures and Tables

**Table 1:** Patterns of amino acid changes and mutations in rpoB gene.

Number of isolates (%)	Amino acid changes in rpoB gene	MTBDRplus assay RIF mutation patterns (rpoB gene)	Finding
14 (20.3%)	No change	WT	RIF^s^
2 (2.9%)	Not known	Δ WT8	RIF^r^
44 (63.8%)	rpoBS531L (Ser-Leu)	Δ WT8, MUT3	RIF^r^
3 (4.3%)	Not known	ΔWT7	RIF^r^
2 (2.9%)	rpoBH526Y (His-Tyr)	ΔWT7, MUT2A	RIF^r^
1 (1.4%)	Not known	ΔWT5, 6	RIF^r^
3 (4.3%)	rpoBD516V (Asp-Val)	Δ WT3,4, MUT1	RIF^r^

**Table 2:** Patterns of amino acids changes and mutations in katG gene.

Number of isolates (%)	Amino acid changes in katG gene	MTBDRplus assay INH mutation patterns (katG gene)	Finding
49 (71%)	katGS315T1 (Ser-Thr)	ΔWT, MUT1	INH^r^
2 (2.9%)	Not known	ΔWT	INH^r^
18 (26.1%)	No change	WT	INH^s^

**Table 3:** Patterns of amino acid changes and mutations on inhA gene.

Number of isolates (%)	Nucleotide/amino acid changes on inhA gene	MTBDRplus assay INH mutation patterns (inhA gene)	Finding
5 (7.2%)	inhAC15T	ΔWT1, MUT1	INH^r^
1 (1.4%)	inhAT8C	ΔWT2, MUT3A	INH^r^
63 (91.3%)	No change	WT	INH^s^

**Table 4:** Comparison of genotype MTBDRplus assay with conventional drug susceptibility testing for identification of drug resistant isolates.

Genotype MTBDRplus assay	Conventional drug susceptibility test				
	INH and RIF resistant	All drugs resistant	Only RIF sensitive	All drugs sensitive	Total
Wild type	3	4	0	5	12
INH resistant, RIF sensitive	0	0	2	0	2
RIF resistant, INH sensitive	0	0	0	0	0
INH and RIF resistant (MDR)	32	23	0	0	55

Total	35	27	2	5	69

## Abbreviations

MDR-TB: Multidrug-resistant tuberculosis
DOTS: Directly observed treatment short course
WHO: World health organization
RIF: Rifampicin
INH: Isoniazid
DNA: Deoxyribonucleic acid
PCR: Polymerase chain reaction
LiPAs: Line-probe assays
XDR-TB: Extensively drug-resistant tuberculosis
DNA: Deoxyribonucleic acid.
